# Clonal dominant grass *Leymus chinensis* benefits more from physiological integration in sexual reproduction than its main companions in a meadow

**DOI:** 10.3389/fpls.2023.1205166

**Published:** 2023-08-11

**Authors:** Jian Guo, Haiyan Li, Yunfei Yang, Xuechen Yang

**Affiliations:** ^1^ School of Environmental Engineering, Xuzhou University of Technology, Xuzhou, China; ^2^ Institute of Grassland Science, Key Laboratory of Vegetation Ecology of the Ministry of Education, Jilin Songnen Grassland Ecosystem National Observation and Research Station, Northeast Normal University, Changchun, China; ^3^ State Key Laboratory of Black Soils Conservation and Utilization, Northeast Institute of Geography and Agroecology, Chinese Academy of Sciences, Harbin, China

**Keywords:** dominant species, companion species, perennial herb, resource translocation, sexual reproduction, tillering node connection, relative benefit

## Abstract

The bioecological characteristics of plants determine their status and role in the community. The advantages of dominant species in the community compared with companion species in terms of physiological and ecological characteristics remain unclear. When both dominant and companion species in grassland plant communities are clonal, these plants are able to share resources within clones (physiological integration). However, it is unclear how the clonal dominant and companion species differ in the effect of their physiological integration on sexual reproduction. We chose *Leymus chinensis*, the dominant species of the most widespread meadow plant communities in the semiarid and arid regions of northern China, and its main companion species *L. secalinus*, *Calamagrostis ripidula*, *C. pseudophragmites*, and *C. epigeios* and conducted a series of *in situ* field experiments in a homogeneous environment, including the determination of the phenotypic characteristics of reproductive ramets with connected (allowing physiological integration) and disconnected (preventing integration) tillering nodes for each species, as well as ^15^N leaf labeling of ramet pairs at the milk-ripe stage. In the clonal populations of the five grasses, physiological integration between vegetative ramets and reproductive ramets interconnected by tillering nodes significantly increased the leaf, stem, inflorescence and ramet biomasses of reproductive ramets, and relative changes in ramet biomass were greatest in *L. chinensis*. ^15^N labeling showed that vegetative ramets supplied nutrients to reproductive ramets through tillering nodes; the amount of translocated ^15^N per unit of reproductive ramet biomass was highest in *L. chinensis*. Overall, our results indicate that in the five clonal grasses, physiological integration between functionally different ramets under tillering node connections had a significant positive effect on sexual reproduction, indicating interspecific consistency in the contribution of physiological integration to sexual reproduction between the dominant and companion species, but this positive effect was greater in the dominant species *L. chinensis* than in the four main companion species. Therefore, differences in the physiological integration ability between the dominant and main companion species, identified for the first time in this study, may explain, at least partly, the dominance of *L. chinensis* in the community.

## 1 Introduction

Plants rarely grow alone and often cluster together to form communities in nature. Based on the different statuses and roles of species in the community, they can be classified as dominant species, companion species and other types. Dominant species are species that have a high abundance relative to that of other species in a community and have obvious control over the community structure and environmental conditions, and their status and development trend in the community largely influence the stability and species diversity of the community (Smith and Knapp, 2003; Avolio et al., 2019). By contrast, companion species are species that occur frequently in the community and exist in companion with the dominant species but do not have major effects on the community structure or environmental conditions (Yang and Zhu, 2011). Although it is now recognized that dominant species in any community have a significant competitive advantage over their companion species, which is closely related to their large number of individuals and high biomass, it is surprising that relatively few studies have been conducted on the physiological and ecological characteristics of dominant and companion species in communities. In forest plant communities, dominant species in the arboreal layer showed significant superiority in the maximum net photosynthetic rate per unit leaf area (Zhang et al., 2017) and specific leaf area (Wang et al., 2014) compared with those of companion species. In grassland plant communities, both dominant and companion species are capable of clonal growth. However, little is known about whether clonal dominant species have physiological and ecological characteristics superior to those of companion species.

An important and unique feature of clonal plants is physiological integration (intraclonal resource sharing), i.e., the translocation of resources such as water, mineral nutrients and carbohydrates between connected ramets of the same clone (Ashmun et al., 1982; Alpert, 1996). For dominant plant species, physiological integration has been repeatedly shown to promote the establishment of newly produced daughter ramets (Evans and Cain, 1995; Dong and Alaten, 1999; Sun et al., 2022a), to increase the growth of ramets in stressful or heterogeneous environments (Roiloa et al., 2014; Zhou et al., 2014), and to enhance the fitness of the whole clone (Song et al., 2013; Chen et al., 2015). Other studies have shown that physiological integration can also greatly increase the growth performance of cooccurring plant species (Wang et al., 2017a; Roiloa et al., 2019; Zhang et al., 2022). Most previous studies on physiological integration focused on the survival and growth of clonal plants. Sexual reproduction is an important link in the life history of clonal plants (Eriksson, 1997), an important means of their adaptation to unstable environments and the geographical migration of species (Eckert, 2001), and therefore, it is vital to population adaptation and evolution. However, it is poorly understood how physiological integration affects sexual reproduction in clonal plants and whether there are differences in the physiological integration ability between clonal dominant and companion species.

The nonzonal vegetation (i.e., meadows) in the semiarid and arid regions of northern China is an important part of the terrestrial ecosystem of the Eurasian steppe and is also the most widely distributed natural vegetation. The dominant species of meadow plant communities is perennial rhizomatous *Leymus chinensis*, and the common main companion species are *L. secalinus*, *Calamagrostis ripidula*, *C. pseudophragmites*, and *C. epigeios* (Li et al., 2001); they are all typical clonal plants of Gramineae. *L. chinensis* has high nutritional value and good palatability; therefore, different types of *L. chinensis* meadows are excellent mowing and grazing grounds (Jia, 1987; Zhu, 2004). Because of its economic and ecological significance, *L. chinensis* has received considerable attention (Guo et al., 2021; Meng et al., 2022; Sun et al., 2022b). However, its main companion species, such as *L. secalinus*, *C. ripidula*, *C. pseudophragmites* and *C. epigeios*, have received less attention. For example, in terms of physiological integration between connected ramets, Gao et al. (2014) found that physiological integration increased the ramet biomass of *L. chinensis* in environments with resource heterogeneity, and Zhou et al. (2014) reported that physiological integration increased the maximum net photosynthetic rate, apparent quantum efficiency, respiration rate, water use efficiency, and chlorophyll content of *L. chinensis* in environments with nutrient heterogeneity and confirmed that differences in the physiological integration ability between the two ecotypes resulted in their different performance levels. Sui et al. (2011) found that physiological integration enhanced the total biomass, belowground biomass, ramet number and total rhizome length of *L. secalinus* under mechanical stimulation. No studies on physiological integration have been reported thus far in *C. ripidula*, *C. pseudophragmites* and *C. epigeios*. Therefore, it is unclear how physiological integration affects sexual reproduction in *L. chinensis* and its four main companion species as well as whether there are interspecific differences in the physiological integration ability.

In this study, we grew *L. chinensis* and its main companion species *L. secalinus*, *C. ripidula*, *C. pseudophragmites* and *C. epigeios* in a homogeneous field environment and measured the phenotypic characteristics of reproductive ramets with connected (allowing physiological integration) and disconnected (preventing integration) tillering nodes for each species. We also labeled the vegetative ramets with an isotope (^15^N) at the milk-ripe stage to verify whether vegetative ramets translocated resources toward the connected reproductive ramets. The objectives of our study were (1) to assess the effect of physiological integration on the sexual reproductive performance of the dominant species *L. chinensis* and its four main companion species and (2) to explore the differences in the physiological integration ability among the dominant species *L. chinensis* and its four main companion species. Here, we hypothesize that (1) physiological integration will increase the sexual reproductive performance of the dominant species *L. chinensis* and its four main companion species and (2) the positive effect of physiological integration will be greater in the dominant species *L. chinensis* than in its four main companion species.

## 2 Materials and methods

### 2.1 Study area

This study was conducted at the Jilin Songnen Grassland Ecosystem National Observation and Research Station (44°38′N, 123°41′E), which is in the southern region of the Songnen Plain. This area has a semiarid, semihumid, and temperate continental monsoonal climate with rainy, hot summers and dry, cold winters. The annual mean temperature ranges from 4.6°C to 6.4°C, and the annual mean precipitation varies from 300 mm to 450 mm, with the majority concentrated from June to September. The growing season with a frost-free period is approximately 130-165 days (Li et al., 2018; Guo et al., 2020a). The meadow vegetation in this study area is dominated by *L. chinensis*, which is accompanied by *L. secalinus*, *C. ripidula*, *C. pseudophragmites*, *C. epigeios*, *Hierochloe glabra*, etc.

### 2.2 Study species

A total of five species were included in this study, namely, *L. chinensis*, *L. secalinus*, *C. ripidula*, *C. pseudophragmites* and *C. epigeios*, all of which are main forage grasses in natural grassland. *L. chinensis* is a perennial grass that is widely distributed in western North Korea, the People’s Republic of Mongolia, northwestern Siberia, the Inner Mongolian Plateau, and the Northeast Plain of China (Kuo, 1987). *L. chinensis* has very strong adaptability and tolerance to saline-alkaline, drought and low-temperature conditions (Jin et al., 2008; Chen et al., 2013; Zhai et al., 2014); thus, it often forms *L. chinensis* steppes and meadows as a dominant species, among which *L. chinensis* meadows are the most widely distributed natural vegetation in the study area. *L. secalinus* is a perennial grass that mainly occurs in typical steppe, sandy grassland, mountain slope, farmland and roadside habitats in northern China, Korea and Japan (Dong, 1999). *L. secalinus* has strong tolerance to drought, low temperature, and light soil salinization. *C. ripidula*, *C. pseudophragmites* and *C. epigeios* are all perennial species that occur in natural grasslands, artificial forest edges and understories in temperate regions of Eurasia and are tolerant to saline-alkaline conditions and certain humidities (Jia, 1987). These five grasses are all typical clonal plants; the clonal ramets are interconnected via rhizomes or tillering nodes (which refer to the unelongated basal internodes of ramets). It is common for the five grasses to have one reproductive ramet and one vegetative ramet per tillering node. On the Songnen Plain, the five grasses usually begin turning green in April, undergo heading in May-June, and then flower and fruit in June-August (Zhu, 2004).

### 2.3 Experimental platform

A total of 75 experimental plots were established at the beginning of May 2017. Each plot had an area of 1 m^2^ (1 m × 1 m), and adjacent plots were at least 0.5 m apart. For each species, vegetative ramets of a similar size were collected from fifteen populations that were 50 m apart from each other in the natural meadow, and thus, each species was represented by fifteen clones (genotypes). We then adopted a completely randomized experimental design and transplanted nine vegetative ramets of any one species in each plot with rows 0.25 m apart and ramets 0.25 m apart. All of the experimental plots were manually irrigated for several days after transplanting to ensure that the ramets survived. Then, the plots were weeded regularly, without any irrigation or fertilization, and the five species were not affected by any pests or diseases. The soil type is sandy loam (Li et al., 2001; Guo et al., 2020a). The soil of the top 30-cm-thick layer was homogenous, and the total N content, total organic C content and total P content were 1.05 ± 0.01 g kg^-1^, 5.82 ± 0.09 g kg^-1^, and 0.76 ± 0.01 g kg^-1^, respectively. The pH was 8.34 ± 0.04, the bulk density was 1.18 ± 0.02 g cm^-3^, and the electrical conductivity was 75.03 ± 0.80 μS cm^-1^.

### 2.4 Intact and severed ramet design

**Figure 1:**
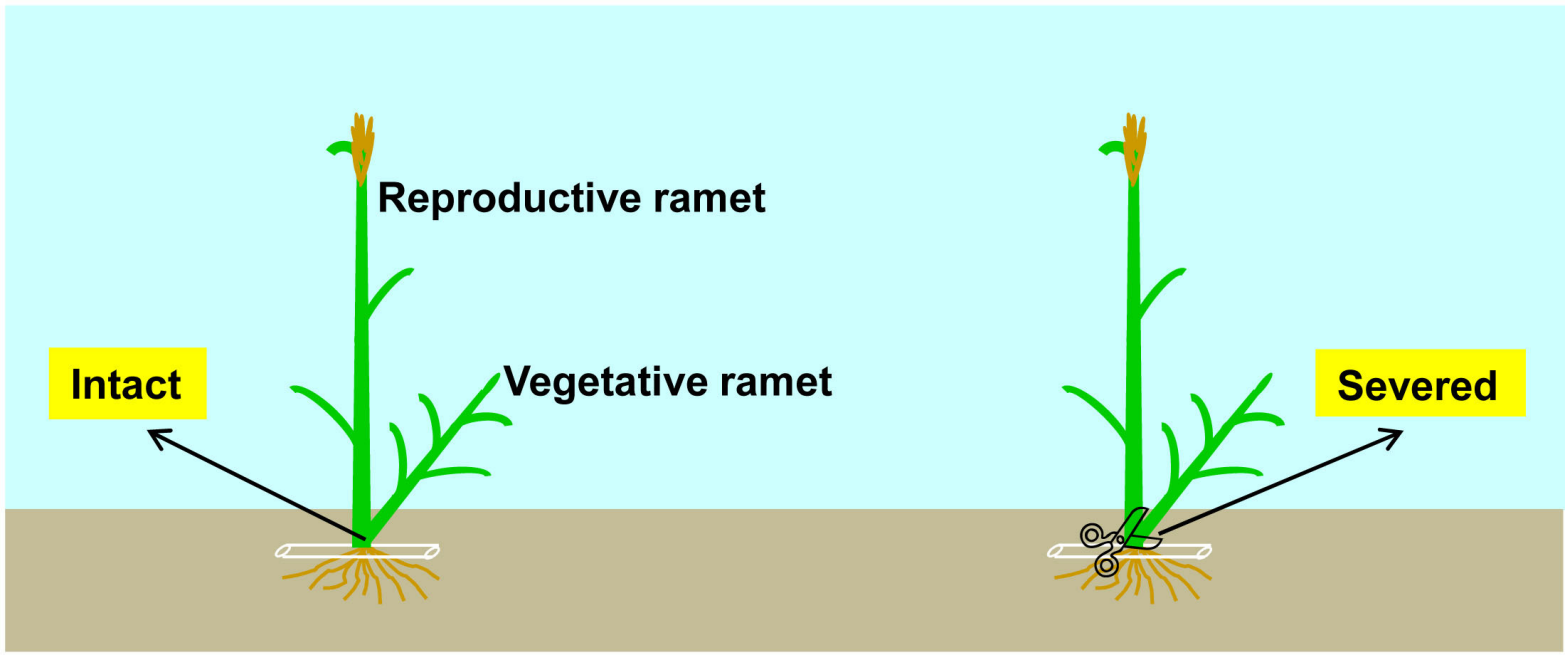
Schematic representation of the experimental design. Each clonal fragment of the five clonal plant species (*Leymus chinensis*, *L. secalinus*, *Calamagrostis ripidula*, *C. pseudophragmites* and *C. epigeios*) consisted of one reproductive ramet and one vegetative ramet. Physiological integration: connections between the reproductive ramet and vegetative ramet remained intact (allowing integration) (left) or were severed (preventing integration) (right).

To investigate the effect of physiological integration on sexual reproduction as well as its interspecific differences, at the early heading stage of each species in both 2018 and 2019, we used colored tags to mark two ramet pairs with similar sizes at the edge of each plot. Each ramet pair consisted of one reproductive ramet (mother ramet) and one vegetative ramet (daughter ramet) connected by a tillering node, and the inflorescence top of the reproductive ramet reached approximately 1 cm above the flag leaf sheath. The connection between the reproductive and vegetative ramets of one ramet pair was left intact (allowing integration), while the connection between the reproductive and vegetative ramets of the other ramet pair was severed (preventing integration) with scissors (**Figure 1**). We gently set aside the topsoil around the ramet pairs to expose the tillering nodes of the reproductive ramets to verify that the vegetative ramets were indeed growing on the tillering nodes of the reproductive ramets, then cut the vegetative ramets from the tillering nodes with scissors, and finally, rapidly restored the topsoil to its original position. Although the severing of physical connections between the ramets may cause physiological stress and make the plants more susceptible to disease infection (Jónsdóttir and Watson, 1997), we did not observe any signs of disease infection in the reproductive ramets throughout the experiment, so severing the vegetative ramets did not cause harm to the growth of the reproductive ramets. We transported the cutoff vegetative ramets to the laboratory. All marked ramet pairs were harvested at the seed-maturing stage of each species. The reproductive ramet height and inflorescence length were measured. Each reproductive ramet was separated into the leaves, stem and inflorescence. Leaf biomass, stem biomass, inflorescence biomass, reproductive ramet biomass, and vegetative ramet biomass were measured after drying in an oven at 65°C for 48 h.

### 2.5 Stable isotope labeling

To verify whether vegetative ramets translocate their own resources to the connected reproductive ramets during sexual reproduction as well as their interspecific differences, an *in situ* leaf labeling experiment was carried out at the milk-ripe stage of each species in 2019. We randomly selected four out of fifteen plots for each species to carry out the stable isotope labeling experiment. Two ramet pairs with similar sizes (one ramet pair for the control treatment and another for the ^15^N labeling treatment) were randomly chosen at the edge of each plot, and each ramet pair consisted of one reproductive ramet (mother ramet) and one vegetative ramet (daughter ramet) connected by a tillering node. The two ramet pairs were at least 50 cm apart. The control solution was distilled water, and the ^15^N labeling solution was a solution of urea (made at the Shanghai Research Institute of Chemical Industry, China) with a concentration of 0.02 g·mL^−1^ and a ^15^N abundance of 5.18%. The labeling method strictly followed the scheme of Guo et al. (2020a). The aboveground reproductive ramets in both the control treatment and ^15^N labeling treatment in each plot were harvested exactly 2 days after labeling. Each reproductive ramet was de-enzymed at 105°C for 30 min and then dried at 65°C for 48 h. We then measured the dry mass of each reproductive ramet and ground it to a fine powder with a ball mill (MM 400 Retsch, Haan, Germany). For each sample, approximately 3 mg of solid powder was loaded into a capsule, and then the isotope values (*δ* ^15^N) were determined using a vario EL cube (Elementar, Langenselbold, Germany) interfaced with an Isoprime 100 isotope-ratio mass spectrometer (Elementar, Langenselbold, Germany), with an overall precision greater than 0.2‰. The amount of ^15^N translocated from the labeled vegetative ramets toward the unlabeled reproductive ramets was calculated following the protocol of Guo et al. (2020a).

### 2.6 Statistical analysis

The statistical analysis was performed with IBM SPSS 20.0 (SPSS Inc., Chicago, IL, USA). All variables were tested for a normal distribution and homogeneity of variances. All results were reported as the means ± standard errors, and a significance level of *P* ≤ 0.05 was used for all analyses.

For each species, a paired-samples *t* test was used to determine the differences in ramet height, inflorescence length, leaf biomass, stem biomass, inflorescence biomass and ramet biomass between tillering node connections that remained intact and were severed and to test for differences in the *δ* ^15^N of reproductive ramets between the control and ^15^N labeling treatments. One-way analysis of variance (ANOVA) was performed to assess the effects of species identity on the absolute and relative benefits of reproductive ramets, the inflorescence biomass allocation of reproductive ramets, the vegetative ramet biomass, the total amount of translocated ^15^N, and the amount of translocated ^15^N per unit of reproductive ramet biomass. Duncan’s multiple range test was used to test for significant differences between the means of multiple groups. The absolute benefit of reproductive ramets was calculated as the difference in the reproductive ramet biomass of intact clones and the reproductive ramet biomass of severed clones. The relative benefit of reproductive ramets (expressed as a percentage) was calculated as the ratio of the reproductive ramet biomass difference between the two connection treatments to the reproductive ramet biomass of the severed clone. The inflorescence biomass allocation (expressed as a percentage) was calculated as the ratio of the inflorescence biomass to the reproductive ramet biomass. The amount of translocated ^15^N per unit of reproductive ramet biomass was calculated as the ratio of the total amount of translocated ^15^N to the reproductive ramet biomass.

## 3 Results

### 3.1 Biomass production and growth characteristics of reproductive ramets

**Figure 2:**
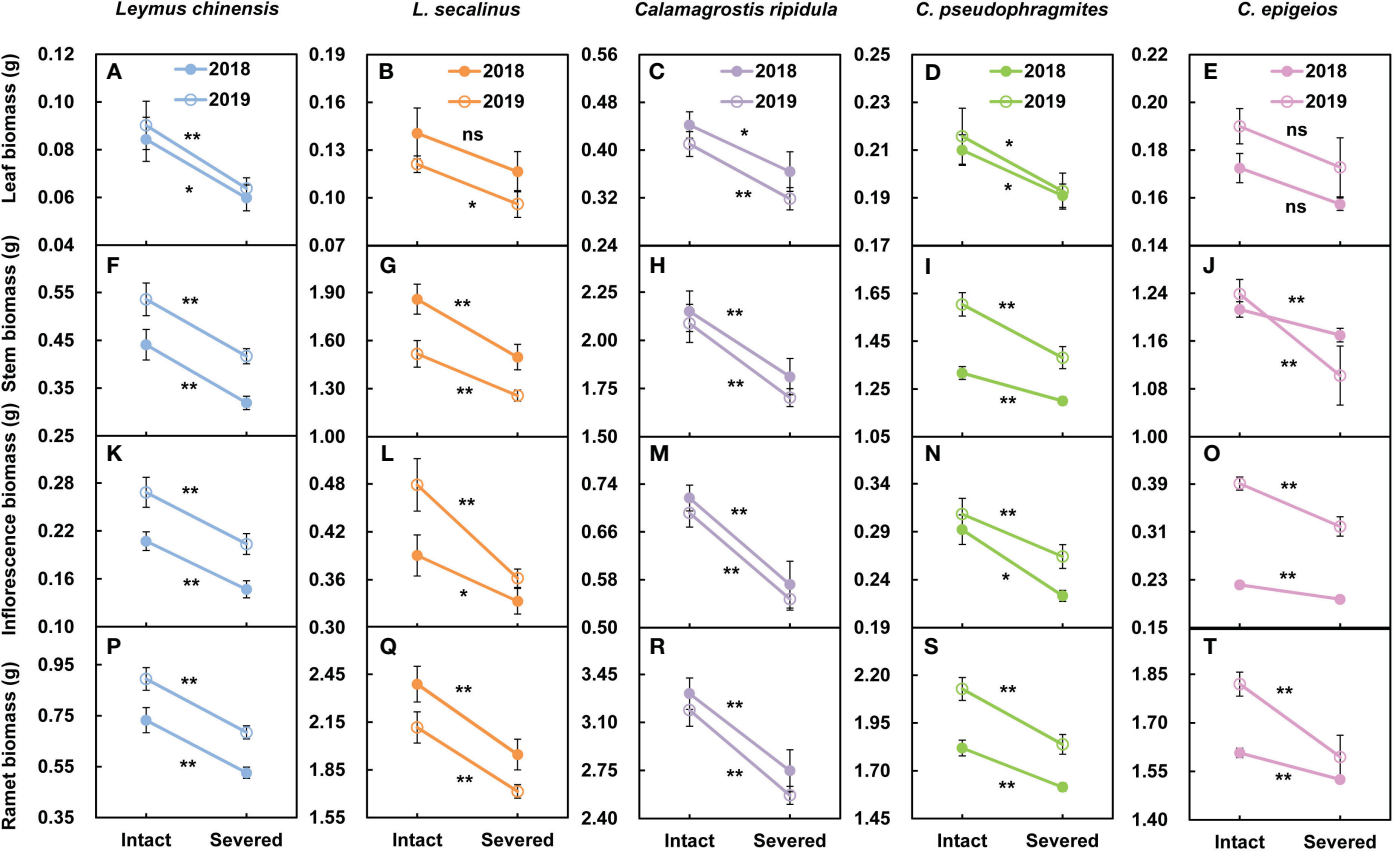
Comparison of the leaf biomass **(A-E)**, stem biomass **(F-J)**, inflorescence biomass **(K-O)** and ramet biomass **(P-T)** of reproductive ramets between the tillering node connections that remained intact and were severed in the five clonal plants grown under the same environmental conditions in 2018 and 2019. Data are means ± SEs (*n* = 15). The *P* values are expressed as follows: **P < 0.01; *0.01 < *P* < 0.05; ns, *P* > 0.05.

In the five clonal plants *L. chinensis*, *L. secalinus*, *C. ripidula*, *C. pseudophragmites* and *C. epigeios*, the leaf biomass (**Figures 2A-E**), stem biomass (**Figures 2F-J**), inflorescence biomass (**Figures 2K-O**) and ramet biomass (**Figures 2P-T**) of reproductive ramets under severed tillering node connections were reduced compared with those under intact connections over the two consecutive years. Except for the leaf biomass of *L. secalinus* in 2018 and the leaf biomass of *C. epigeios* in 2018 and 2019, all differences between the two treatments reached a significant (*P* < 0.05) or extremely significant (*P* < 0.01) level. There was no significant difference (*P* > 0.05) in the height or inflorescence length of reproductive ramets between the tillering node connections that remained intact and severed over the two consecutive years. These results indicated that physiological integration had a significant effect on the biomass production characteristics rather than on the growth characteristics of the reproductive ramets in the five clonal grasses.

### 3.2 Benefit of reproductive ramets

**Figure 3:**
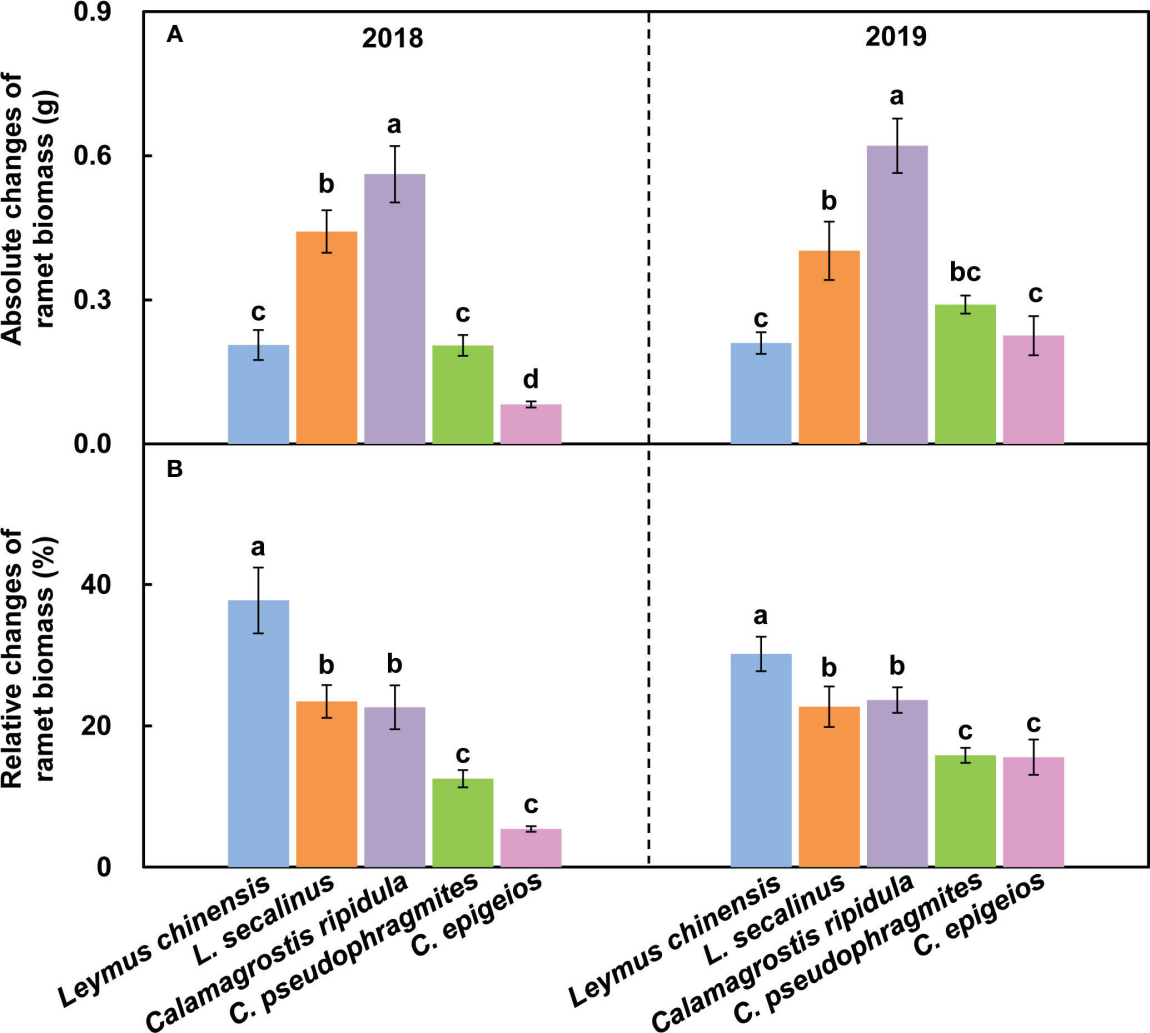
Comparison of the absolute changes in ramet biomass **(A1, A2)** and relative changes in ramet biomass **(B1, B2)** among the five clonal plants in 2018 and 2019. Data are means ± SEs (*n* = 15). Different lowercase letters indicate significant differences (*P* < 0.05) between plant species.

The absolute change in the reproductive ramet biomass of *C. ripidula* was significantly (*P* < 0.05) larger than that of the other four clonal plants over the two consecutive years (**Figures 3A1, A2**). The relative change in the reproductive ramet biomass of *L. chinensis* was significantly (*P* < 0.05) larger than that of the other four clonal plants, while the relative changes in the reproductive ramet biomass of *C. pseudophragmites* and *C. epigeios* were significantly (*P* < 0.05) smaller than those of the other three clonal plants over the two consecutive years (**Figures 3B1, B2**).

### 3.3 Inflorescence biomass allocation of reproductive ramets

**Figure 4:**
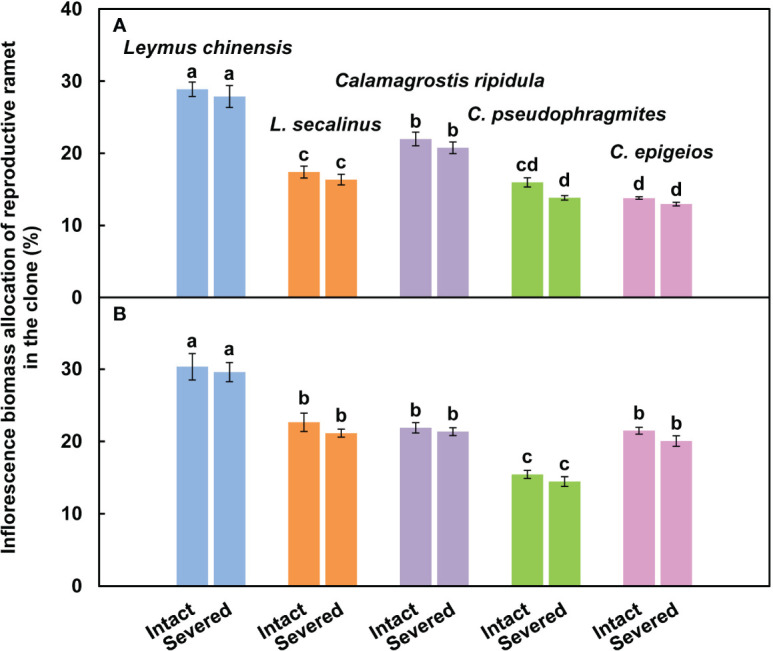
Comparison of the inflorescence biomass allocation of reproductive ramets among the five clonal plants in the intact and severed treatments in 2018 **(A)** and 2019 **(B)**. Data are means ± SEs (*n* = 15). Different lowercase letters indicate significant differences (*P* < 0.05) between plant species.

The inflorescence biomass allocation of the reproductive ramets of *L. chinensis* was significantly (*P* < 0.05) greater than that of the other four clonal plants in both severed and intact clones over the two consecutive years (**Figure 4**).

### 3.4 Biomass production of vegetative ramets

**Figure 5:**
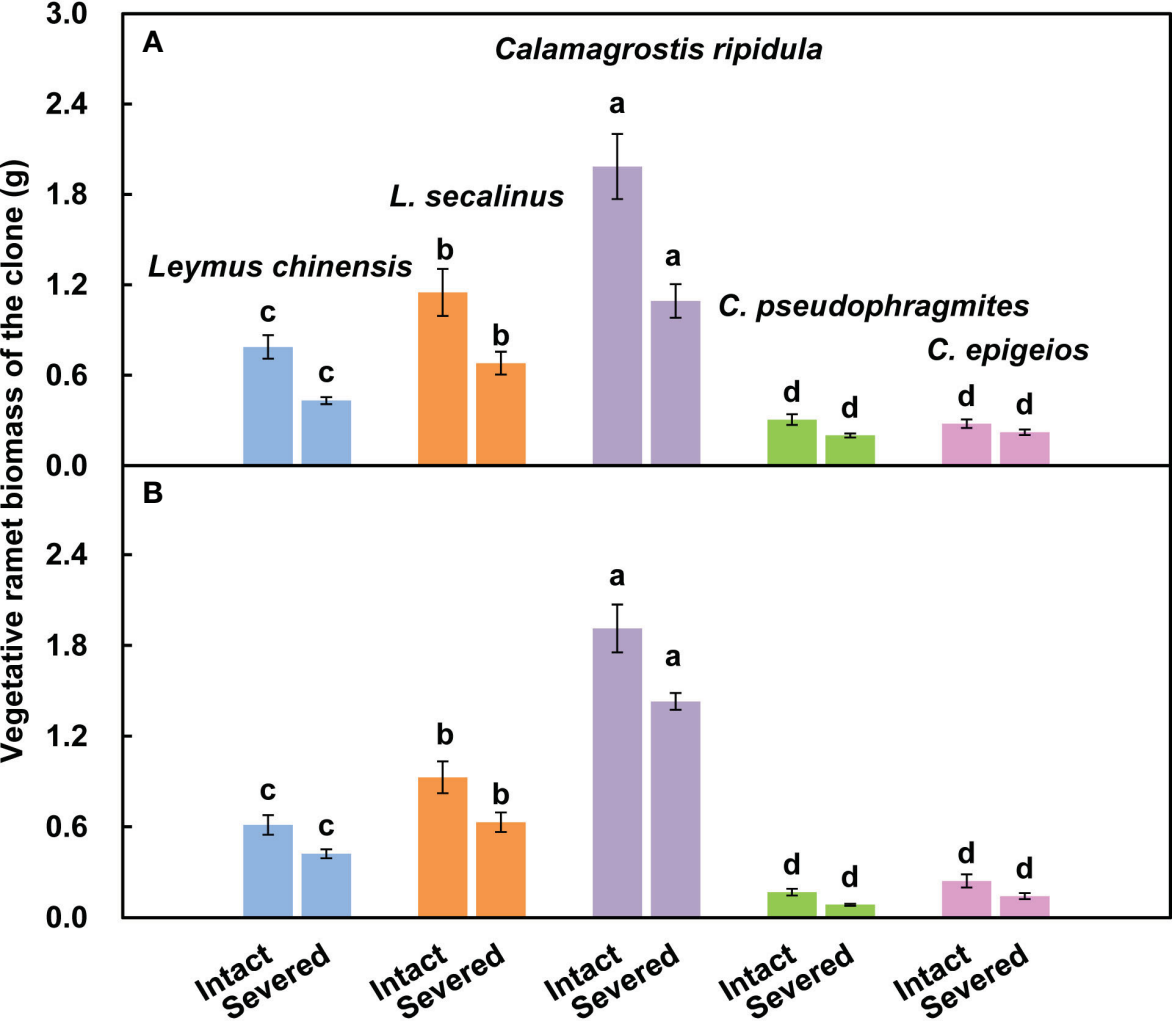
Comparison of the vegetative ramet biomass of clones among the five clonal plants in the intact and severed treatments in 2018 **(A)** and 2019 **(B)**. Data are means ± SEs (*n* = 15). Different lowercase letters indicate significant differences (*P* < 0.05) between plant species.

The vegetative ramet biomass of *C. ripidula* was significantly (*P* < 0.05) larger than that of the other four clonal plants, while the vegetative ramet biomass of *C. pseudophragmites* and *C. epigeios* was significantly (*P* < 0.05) smaller than that of the other three clonal plants in both severed and intact clones over the two consecutive years (**Figure 5**).

### 3.5 Transfer of ^15^N from vegetative ramets to connected reproductive ramets

In the five clonal plants *L. chinensis*, *L. secalinus*, *C. ripidula*, *C. pseudophragmites* and *C. epigeios*, reproductive ramets had a significantly higher *δ* ^15^N level in the ^15^N labeling treatment than in the control treatment (**Table 1**). These results demonstrated that vegetative ramets connected to tillering nodes translocated their own nutrients toward reproductive ramets through physiological integration. The total amount of translocated ^15^N in the reproductive ramets of *L. secalinus* and *C. ripidula* was larger than that of the other three clonal plants, while the total amount of translocated ^15^N in the reproductive ramets of *C. epigeios* was smaller than that of the other four clonal plants (**Table 2**). The amount of translocated ^15^N per unit of reproductive ramet biomass of *L. chinensis* was larger than that of the other four clonal plants (**Table 2**).

**Table 1:** Comparison of the *δ* ^15^N of reproductive ramets between the control and ^15^N labeling treatments in the five clonal plants grown under the same environmental conditions in 2019 (means ± SEs, *n* = 4).

Species	CK	^15^N labeling	*t*	*P*
*Leymus chinensis*	2.37 ± 0.19	125.26 ± 6.80	-17.63	<0.001
*L. secalinus*	3.51 ± 0.44	78.73 ± 5.38	-13.86	0.001
*Calamagrostis ripidula*	4.07 ± 0.63	92.06 ± 2.11	-39.44	<0.001
*C. pseudophragmites*	5.71 ± 0.67	122.75 ± 4.05	-30.85	<0.001
*C. epigeios*	3.01 ± 0.29	66.24 ± 6.69	-9.61	0.002

**Table 2:** Comparison of the amount of translocated ^15^N of reproductive ramets among the five clonal plants grown under the same environmental conditions in 2019 (means ± SEs, *n* = 4).

Species	Total ^15^N amount (μg)	^15^N amount per unit ramet biomass (μg g^-1^)
*Leymus chinensis*	5.50 ± 0.15 bc	7.56 ± 0.53 a
*L. secalinus*	16.37 ± 1.46 a	4.66 ± 0.48 b
*Calamagrostis ripidula*	13.50 ± 0.74 a	3.65 ± 0.33 bc
*C. pseudophragmites*	7.53 ± 0.46 b	3.03 ± 0.13 cd
*C. epigeios*	3.75 ± 0.52 c	2.14 ± 0.35 d

Different lowercase letters indicate significant differences (*P* < 0.05) between different plant species.

## 4 Discussion

### 4.1 Physiological integration increases the sexual reproductive performance of five clonal plants

In grassland plant communities, many plant species are clonal, and one of their unique traits is physiological integration, i.e., the translocation and sharing of resources between ramets through physical connections (Ashmun et al., 1982; Alpert, 1996). First, from the perspective of ramet function (rather than the developmental age of the ramet), two different types of aboveground ramets are widely distributed in clonal plant populations: reproductive ramets and vegetative ramets (Harper, 1977). Therefore, the so-called paired ramet system often includes the following three cases: vegetative ramet–vegetative ramet, reproductive ramet–reproductive ramet, and vegetative ramet–reproductive ramet. Obviously, the first two cases are paired ramet systems with the same function, and the third one is a paired ramet system with different functions. Second, from the perspective of physical connection, apart from rhizome or stolon connections, tillering node connection is also a very common connection form between clonal ramets. Under natural conditions, vegetative ramets may grow on the unelongated basal internodes of reproductive ramets, in which case the two types of ramets are interconnected by tillering nodes. Studies have repeatedly revealed that physiological integration between vegetative ramets and vegetative ramets with the same function via a rhizome or stolon has a positive effect on the survival and growth of the ramets (Evans and Cain, 1995; Dong and Alaten, 1999; Song et al., 2013; Chen et al., 2015), but little research has been performed on the effect of physiological integration between vegetative ramets and reproductive ramets with different functions by tillering node connection on sexual reproductive performance. Our previous studies showed that physiological integration between vegetative and reproductive ramets connected by a tillering node increased sexual reproductive characteristics such as inflorescence biomass, floret number, seed number, seed biomass and the seed-setting rate in *H. glabra* (Guo et al., 2020b) and *L. chinensis* (Guo et al., 2020c). In the present study, by severing connections, we found that physiological integration between vegetative ramets and reproductive ramets connected by a tillering node significantly increased the leaf biomass, stem biomass, inflorescence biomass, and ramet biomass of reproductive ramets in *L. chinensis*, *L. secalinus*, *C. ripidula*, *C. pseudophragmites* and *C. epigeios* (**Figure 2**), and these results supported our first hypothesis. Most importantly, the consistent finding that vegetative ramets are very advantageous for sexual reproduction under tillering node connection in these five clonal plants not only indicates interspecific consistency in the contribution of physiological integration to sexual reproduction in clonal grasses but also reflects the convergent adaptation of physiological integration between the two types of ramets to the same habitat conditions in these five grasses. Our previous research revealed that compared with reproductive ramets with different numbers of connecting vegetative ramets, reproductive ramets with zero connecting vegetative ramets in *H. glabra* had poorer performance, and their inflorescence biomass was significantly lower than that of reproductive ramets with 1, 2, and 3 connecting vegetative ramets (Guo et al., 2020b). These results all imply that vegetative ramets play an important role in sexual reproduction apart from the spatial expansion of clonal plant populations.

The degree to which physiological integration affects sexual reproductive performance varies with reproductive characteristics. In this study, we found that physiological integration had no effect on ramet height or inflorescence length but had significant effects on biomass production characteristics such as leaf biomass, stem biomass, inflorescence biomass and ramet biomass (**Figure 2**), which may be due to the different growth properties and growth times of each phenotypic characteristic. Under natural conditions, the reproductive ramets of the five clonal grasses in this study area usually stop increasing in ramet height and inflorescence length before flowering (*L. chinensis*: mid-June, *L. secalinus*: late June, and *C. ripidula*, *C. pseudophragmites* and *C. epigeios*: early July), and the increases in leaf biomass, stem biomass, inflorescence biomass and ramet biomass will continue until seed maturity (*L. chinensis*: mid-July, *L. secalinus*: late July, and *C. ripidula*, *C. pseudophragmites* and *C. epigeios*: early August). Thus, phenotypic characteristics such as ramet height and inflorescence length are less affected by physiological integration during growth because of the short growth period, whereas phenotypic characteristics related to biomass production are greatly affected by physiological integration because of the long growth period.

Previous studies concerning the effects of the physiological integration of clonal plants were mostly conducted using greenhouse pot experiments (Zhou et al., 2014; Wang et al., 2017a; Zhang et al., 2022), which enable easy manipulation but do not readily result in vegetative ramet–reproductive ramet pairs that meet the experimental requirements due to growth space, growth period and microenvironmental limitations. Field experiments can provide a more realistic test than greenhouse pot experiments but are more difficult to conduct. In this study, we grew five clonal grasses in a homogeneous field environment, and then at the early heading stage of each species, we selected similarly sized, synchronously heading ramet pairs (vegetative ramet–reproductive ramet) as experimental samples, thus minimizing or eliminating the effects of inherent differences and asynchronous heading on sexual reproductive performance (Li et al., 2018). Moreover, the selected ramet pairs were all located at the edges of the plots. Considering the low density of ramets at the edge, the large interval between ramets, and the proximity of resource supply, the other ramets had minimal influence on sexual reproductive performance compared with vegetative ramets growing on the tillering nodes. Therefore, the field experiments that we conducted in a homogeneous environment provide a more realistic test for assessing the effect of physiological integration between different functional ramets connected by tillering nodes on sexual reproductive performance. The effect of physiological integration between different functional ramets connected through rhizomes or stolons on sexual reproductive performance in a heterogeneous environment can be explored in the future.

Isotope labeling technology is an effective means of exploring resource transfer between connected ramets of clonal plants (Zhang et al., 2002). A study using ^15^N isotope labeling revealed that vegetative ramets of the wetland clonal plant *Iris laevigata* could translocate their resources to connected reproductive ramets (Wang et al., 2017b). In this study, when the connected vegetative ramets were labeled with ^15^N at the milk-ripe stage in clonal populations of *L. chinensis*, *L. secalinus*, *C. ripidula*, *C. pseudophragmites* and *C. epigeios* growing in a homogeneous field environment, significantly larger amounts of ^15^N than the background value were detected in the reproductive ramets (**Table 1**), indicating that vegetative ramets can transfer their own resources to the connected reproductive ramets in these five clonal grasses. The same results were obtained in our previous study on *H. glabra* (Guo et al., 2020b). Most importantly, we obtained consistent findings among these five clonal grasses, indicating commonality among them; i.e., vegetative ramets with tillering node connections can supply resources to reproductive ramets through physiological integration at the most critical stage of sexual reproduction, which is beneficial for sexual reproduction, thus further verifying the convergent adaptation of physiological integration between the two types of ramets to the same habitat conditions in the five clonal grasses.

### 4.2 Differences in the capacity for physiological integration between *L. chinensis* and its major companion species

Differences in physiological integration ability among clonal plants may determine their performance differences in the community. Many invasive alien plants are clonal. A comparative study on the physiological integration ability of invasive plants and congeneric co-occurring native plants showed that invasive plants had a higher physiological integration ability than native plants in heterogeneous environments, and physiological integration benefitted invasive clonal plants more than native plants and thus may confer a competitive advantage to invasive plants (Wang et al., 2017a). However, in grassland plant communities, when both dominant species and companion species are clonal, it is not clear whether the native dominant species have a higher physiological integration ability than the companions. In the present study, we found that the absolute change in reproductive ramet biomass was the largest for *C. ripidula* rather than for the dominant species *L. chinensis* (**Figures 3A1, A2**), indicating that the reproductive ramets of *C. ripidula* benefit more from physiological integration in absolute terms than those of the other four grasses. One potential mechanism for the higher absolute benefit of physiological integration in *C. ripidula* may be that this species has a higher capacity for resource translocation from vegetative ramets to their connected reproductive ramets than the other four grasses. We used isotope labeling to detect the amount of resource translocation from vegetative ramets and found that *C. ripidula* had a higher N translocation efficiency (**Table 2**). Another potential mechanism may be that the vegetative ramets of *C. ripidula* can absorb and utilize resources such as nutrients, water and light more efficiently than those of the other four grasses, so that a stronger source of nutrients, water and photosynthates could be created in the vegetative ramets of *C. ripidula* than in those of the other four grasses. We found that the vegetative ramet biomass of *C. ripidula* was significantly larger than that of the other four grasses in both severed and intact clones (**Figure 5**), confirming the plausibility of the second mechanism. Both a higher resource translocation capacity and a stronger source would allow for higher resource translocation from the vegetative to the reproductive ramets and thus benefit the growth of the latter.

Irrespective of the effect of physiological integration, we also found that *C. ripidula* generally produced taller and heavier reproductive ramets than the other four grasses (**Figure 2**). Our finding that reproductive ramets of *C. ripidula* benefitted more from physiological integration in absolute terms than those of the other four grasses in a homogeneous environment (**Figures 3A1, A2**) could therefore simply indicate that large plants benefit more from physiological integration in absolute terms than small plants in terms of sexual reproduction. In fact, an additional analysis showed that the relative change in reproductive ramet biomass was significantly larger in *L. chinensis* than in the other four grasses (**Figures 3B1, B2**). Meanwhile, isotope labeling showed that the amount of translocated ^15^N per unit of reproductive ramet biomass was significantly larger in *L. chinensis* than in the other four grasses (**Table 2**). Both results indicate that the reproductive ramets of the dominant species *L. chinensis* benefit more from physiological integration in relative terms than those of the other four grasses, which supports our second hypothesis. The total amount of translocated ^15^N or the absolute change in reproductive ramet biomass is mainly caused by inherent differences in biology between species, while the amount of translocated ^15^N per unit of ramet biomass or the relative change in reproductive ramet biomass better reflects differences in physiological integration ability and their effects between species. Thus, the greater physiological integration ability of *L. chinensis* may confer an advantage over its four companion species in terms of sexual reproduction. This study is the first to report differences in the physiological integration ability of these five clonal grasses.

### 4.3 Analysis of the comprehensive reasons for *L. chinensis* being a dominant species in the meadow plant community

The bioecological characteristics of plant species determine their status and role in the community. Their tolerances to adversity, vegetative propagation characteristics, sexual reproduction characteristics, and physiological characteristics are important manifestations of their bioecological characteristics. *L. chinensis* is the dominant species of the most widely distributed natural meadow vegetation in the study area, while *L. secalinus*, *C. ripidula*, *C. pseudophragmites* and *C. epigeios* are the common companion species (Li et al., 2001). On the one hand, *L. chinensis* is more tolerant to drought and saline-alkaline conditions in representative habitats in the study area than *L. secalinus*, *C. ripidula*, *C. pseudophragmites* and *C. epigeios* (Jia, 1987). On the other hand, the tillering nodes of *L. chinensis* ramets can propagate for four generations (Yang et al., 2003), while those of *L. secalinus* propagate for only two generations (Yang and Zhang, 2004) during the growing season in sandy soil habitats with enough growing space and no interspecies competition, implying that the tillering nodes of *L. chinensis* have a higher capacity for vegetative propagation than those of *L. secalinus*. In long-term mowed meadows, the clonal ramets of *L. chinensis* populations consisted of four age classes (Zhang et al., 2020), while the clonal ramets of *C. ripidula* (Yang et al., 1998), *C. pseudophragmites* (Yang and Zheng, 2000) and *C. epigeios* (Zhang et al., 2016) populations all consisted of two age classes, indicating that the clonal ramets of *L. chinensis* have a higher capacity for vegetative propagation than those of *C. ripidula*, *C. pseudophragmites* and *C. epigeios*. Therefore, these findings explain why *L. chinensis* became the dominant species, while *L. secalinus*, *C. ripidula*, *C. pseudophragmites* and *C. epigeios* became the companion species in the community from the perspective of vegetative propagation.

Sexual reproductive capacity affects the competitiveness of plant species in the community and their adaptability to changing environments. In this study, although the inflorescence biomass and ramet biomass of the reproductive ramets of *L. chinensis* were lower than those of the other four grasses (**Figure 2**), the inflorescence biomass allocation was significantly greater than that of the other four grasses (**Figure 4**). Allocation to sexual reproduction in *L. chinensis* is more advantageous than that in the other four grasses. Previous studies also showed that the inflorescence biomass allocation of reproductive ramets of *L. chinensis* was significantly greater than that of *L. secalinus* (Hong, 2020), *C. ripidula*, *C. pseudophragmites* and *C. epigeios* (Li, 2002) in this study area. In addition, we found that the amount of translocated ^15^N per unit of reproductive ramet biomass was significantly larger in *L. chinensis* than in the other four grasses (**Table 2**), which means that the physiological integration ability and its effects were evidently stronger in *L. chinensis* than in the other four grasses. Therefore, differences in the tolerance, vegetative propagation characteristics, sexual reproduction characteristics, and physiological integration ability of the five clonal grasses together determine their status in the community.

## 5 Conclusions

In clonal populations of the dominant species *L. chinensis* and its main companion species *L. secalinus*, *C. ripidula*, *C. pseudophragmites* and *C. epigeios* growing in a homogeneous field environment, physiological integration between vegetative and reproductive ramets connected by tillering nodes significantly increased the leaf biomass, stem biomass, inflorescence biomass and ramet biomass of reproductive ramets. Vegetative ramets translocated their own resources to the connected reproductive ramets through tillering nodes. The physiological integration ability and its positive effect on sexual reproduction were stronger in the dominant species *L. chinensis* than in the other four main companion species. This study is the first to explain, from the perspective of physiological integration, the dominance of *L. chinensis* and the companion status of *L. secalinus*, *C. ripidula*, *C. pseudophragmites* and *C. epigeios* in the community. However, the current study was carried out in a homogeneous field environment with only one level of resource supply. Further studies that explore the effects of physiological integration between different functional ramets connected through rhizomes under different levels of resource supply or a heterogeneous resource supply will help us better understand the population adaptation and species evolution of clonal dominant species and their companion species in grassland plant communities in future environments.

## Data availability statement

The raw data supporting the conclusions of this article will be made available by the authors, without undue reservation.

## Author contributions

HL and YY designed the experiments. JG performed the experiments. JG and XY analyzed the data. JG and HL wrote the manuscript. All authors read and approved the manuscript. All authors contributed to the article.
